# Association Between Calcium Level, Bone Mineral Density, and Hip Fracture: Analysis Based on NHANES Data

**DOI:** 10.3390/healthcare13040356

**Published:** 2025-02-07

**Authors:** Xiao Wang, Zisheng Ai

**Affiliations:** Department of Medical Statistics, School of Medicine, Tongji University, Shanghai 200331, China; 2111559@tongji.edu.cn

**Keywords:** association, mediation, calcium, bone mineral density, hip fracture, NHANES

## Abstract

**Introduction:** The relationships between calcium, bone mineral density, and hip fracture have been studied for a long time, but there are still different opinions on the matter. The aim of this study was to decipher the relationship between these factors from National Health and Nutrition Examination Survey (NHANES) data. **Methods:** After we performed data cleaning for the obtained NHANES data, we used multiple imputation to obtain the complete data and conducted an analysis for different variables. First, by using multivariate linear regression models, we confirmed the association between calcium and bone mineral density, and then we confirmed the association between bone mineral density and hip fracture by using multivariate logistic regression models. A mediation analysis of these variables was performed. **Results:** The analysis in this study included data on 18,003 participants from the NHANES, and we were able to find a strong association between calcium and bone mineral density (*p* < 0.001). The association between bone mineral density and hip fracture was also significant (*p* < 0.001). One augmented gram of daily calcium intake was associated with a 0.04 unit increase in BMD level, and a one unit increase in BMD level could downgrade the occurrence of hip fracture for 5.4 times. The mediation analysis showed that the femur BMD level and total BMD level have a mediating relationship with hip fracture, and no clear relationship among calcium, BMD, and hip fracture could be established. **Conclusions:** Although it is difficult to draw strict conclusions from the mediation analysis in this study, we can observe a clear association between calcium and BMD as well as an association between BMD and hip fracture.

## 1. Introduction

The global burden of fractures is substantial: in 2017 [1] and 2019 [2], around 360 million and 445 million people, respectively, had fractures worldwide, and the years of healthy life lost due to disability (YLDs) were 19.8 million and 25.8 million years, which represent a great loss for every country. We also know that the prevalence has been increasing every year for decades [1], and the burden is predicted to double by 2040 [3]. Of all the fracture types, hip fractures are well known for their severity and potential to lead to additional fractures [4] and are a common cause of morbidity and mortality [5]. To help improve the treatment and management of hip fractures, efforts have been made by various groups, including the American Academy of Orthopedic Surgeons (AAOS) [6]. The AAOS has made multiple recommendations for the management of hip fractures: osteoporosis evaluation, calcium supplementation, etc.

The bone mineral density (BMD) test is a measure of the amount of calcium and other minerals in bone. The BMD with T-score measurement has long been used to diagnose osteoporosis in clinical practice, and it was found to be associated with fracture [2,7,8,9]. Race, gender, behavior, and diet were also reported having influences on bone mineral density [10]. Results showed that osteoporosis with low BMD significantly increased the risk for any fracture, including hip fracture, and the diagnosis of osteoporosis was recognized as being at an elevated risk for fracture [9,11]. Evidence also showed that a reduction in bone mineral density could result in an increased risk of fractures [12], and specifically, the femoral BMD level could lead to future hip fractures [13].

The serum calcium level could influence susceptibility to hip fracture [14], and studies showed that a sufficient intake of calcium might lead to a low risk of hip fractures [15]. On the other hand, based on the results of a systematic review and meta-analysis study, calcium supplementation was not associated with hip fracture [16]. Some researchers pointed out that although taking calcium might improve BMD levels, it might not reduce the risk of hip fracture, and the study also mentioned that vitamin D level might also impact the results [17]. In contrast, other studies claimed that sufficient calcium supplementation could prevent hip fracture but had no impact on BMD level, but the study used data from different studies with various baseline settings [18].

From the above, although different studies demonstrated that varied possible associations might exist, we can see that the association between calcium, BMD, and hip fracture is not clear yet. These inconsistent results made us feel curious as to whether there is a single source of data that could be used to identify, and then we could find whether the supplementation of calcium should be recommended for preventing hip fracture based on the study result. Thus, in this research, we aimed to evaluate the association of hip fracture with other factors based on the NHANES (National Health and Nutrition Examination Survey) data from a sole source.

## 2. Materials and Methods

### 2.1. Study Population

The objective of this cross-sectional study is to examine the association between calcium, BMD and hip fracture in the U.S. population using data based on NHANES data. The NCHS (National Center for Health Statistics) started the program of the NHANS in the early 1960s, and the survey data include different aspects of risks for people from the entire United States population. The data were collected after a written agreement was signed by a participant, and all the data released publicly on the website were approved by the NCHS Ethics Review Board [19]. Data extracted from NHANES can be also found from the Supplementary Materials.

**Figure 1:**
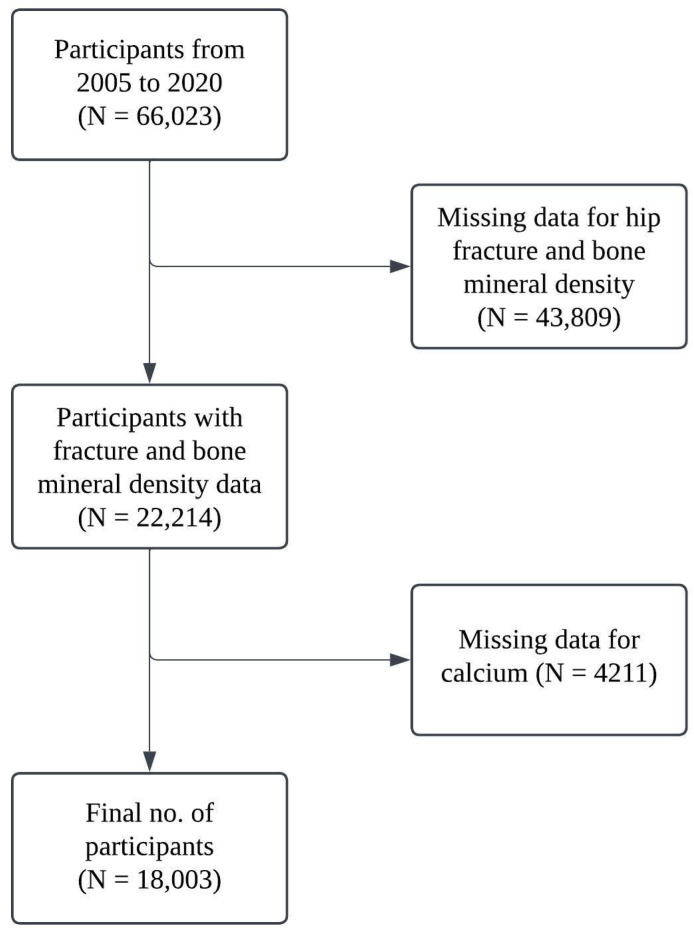
Workflow chart for participants included.

All participants who completed the relevant NHANES survey were included in the study, and participants with missing data on key variables were excluded. In this study, 66,023 participants were enrolled initially in the period from 2005 to 2020, and 43,809 participants have missing data in relation to hip fracture and bone mineral density. Ultimately, 18,003 participants were included in this study with complete data for both calcium intake and total calcium. A workflow chart of the participants is presented in Figure 1.

### 2.2. Daily Intake and Assessment of Calcium

The dietary and supplemental intake for calcium (mg) from separate interviews for two days were added together and divided to obtain the mean, focusing on one variable in this study. The status code of dietary data was used to obtain reliable data for calcium intake; code 1 in the datasets indicates that the data were reliable and met the minimum criteria.

### 2.3. Assessment of BMD and Hip Fracture

In this study, femur scans based on dual-energy X-ray absorptiometry were used to obtain the measurement data for the bone mineral density for the total femur and femoral neck [20]. The scans were acquired on the Hologic Discovery model A densitometers (Hologic, Inc., Bedford, MA, USA), using software version Apex 3.2. The included data were selected from where the scan examination status code is 1, meaning the femur scan was completed and all data were valid. The total femur BMD (gm/cm^2^) and femoral neck BMD (gm/cm^2^) were separately measured in the femur scans. The times of hip fracture were collected in the osteoporosis questionnaire, which was used to establish the status of the hip fracture, i.e., whether or not it happened.

### 2.4. Covariates

Covariates such as gender, age, race, marital status, family poverty income ratio (PIR—ratio of family income to poverty threshold), BMI (body mass index), waist circumference, smoking status, drinking status, and diabetes status were extracted in this study. All missing data for gender (male/female) and age (years) were removed. Race was divided into “Mexican American”, “Non-Hispanic White”, “Non-Hispanic Black”, and “Other Race”. Marital status was divided into “married” and “other”. BMI (kg/m^2^) and waist circumference (cm) were obtained from body measurements. Smoking status and drinking status were divided into “ever” and “other”. Diabetes status was divided into “yes” and “other”.

### 2.5. Statistical Analysis

All analyses were performed with R (version 4.2) [21]. The baseline characteristics between different groups were examined using a *t*-test for continuous variables and a chi-square test for categorical variables. Multivariate logistic regression models were used to check the associations between calcium levels, BMD levels, and hip fracture status [22]. Multiple imputation of missing data was used to impute missing data for covariates [23]. Covariate selection was conducted based on AIC (Akaike information criteria) [24]. Total calcium levels were associated with intake calcium levels, and BMD levels were associated with calcium levels. We treated hip fracture status as a dependent variable and others as independent variables. Mediation analyses were used to confirm the relationship between calcium levels, BMD levels, and hip fracture status [25,26,27]. We examined different models for the analysis, including the following:

1. Calcium to BMD: Calcium intake is the independent variable, and total BMD/femur BMD is the dependent variable.
2. BMD to hip fracture: Total BMD is the independent variable, femur BMD is the mediator, and hip fracture is the dependent variable.
3. Calcium to BMD to hip fracture: Calcium intake is the independent variable, total BMD is the mediator, and hip fracture is the dependent variable.

The above models were designed to confirm the associations between different variables and the proportion of the effect mediated following the establishment of these associations; we conducted a mediation analysis to ascertain the extent and the status of different variables. The proportion of the effect mediated was calculated using the formula (mediation effect/total effect) × 100%. The confidence interval of the meditation effect was calculated using the bootstrap method [21].

## 3. Results

### 3.1. Characteristics of the Study Population

In total, 18,003 subjects were included in this study, and the majority of them did not report a hip fracture. As shown in Table 1, no significant difference could be found in terms of sex, BMI, waist circumference, and drinking status for the different groups of subjects, whereas there were differences between the groups in terms of age, race, marital status, smoking status, and diabetes status.

### 3.2. Association of Calcium with BMD

In this investigation, we treated calcium intake as the independent variable and total BMD/femur BMD as the dependent variable. Table 2 shows that both total BMD and femur BMD are associated with daily calcium intake. While the magnitude of the impact is small for BMD, the *p*-values (<0.001) from both models are significant. The results show that the daily calcium intake is highly associated with the BMD level of the participants.

### 3.3. Association of BMD with Fracture

**Figure 2:**
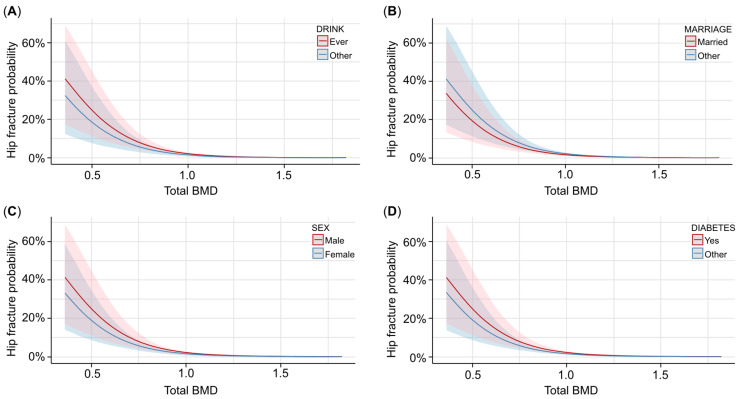
The associations between total BMD and hip fracture probability with different covariates. (**A**) Predicted probability and confidence interval of hip fracture occurrence with different total BMD levels and drinking statuses. Red lines and contours are ever drinking, and blue lines and contours are other drinking statuses. (**B**) Predicted probability and confidence interval of hip fracture occurrence with different total BMD levels and marriage statuses. Red lines and contours are married participants, and blue lines and contours are other participants. (**C**) Predicted probability and confidence interval of hip fracture occurrence with different total BMD levels and genders. Red lines and contours are males, and blue lines and contours are females. (**D**) Predicted probability and confidence interval of hip fracture occurrence with different total BMD levels and diabetes statuses. Red lines and contours are diabetes participants, and blue lines and contours are other participants.

We took total BMD as the independent variable, femur BMD as the mediator, and hip fracture as the dependent variable. Specifically, we selected the covariates using AIC to include the covariates in the model, and then we tested the mediation effect of femur BMD for the same model. For the covariate selection, age, drinking status, PIR, marriage status, sex, diabetes status, and race were included in the model, and the results showed that total BMD had a strong impact on the hip fracture (log[OR] = −5.4; *p* < 0.001). As shown in Table 3, the higher the total BMD, the less likely it was for a hip fracture to occur. Figure 2 shows that for different covariate statuses, the trend between total BMD and hip fracture occurrence probability is similar. From Figure 2, we can see that ever drinking, being unmarried, and male participants with diabetes have a higher risk of hip fracture than their counterparts. We can also see from Figure 2 that different covariates had an impact on the hip fracture occurrence probability, though the influence is not remarkable, especially when the total BMD is large enough, indicating that the covariates have a lower impact when the total BMD level is maintained well.

The mediation effect for the model can be seen in Table 4, and all the effects, including average causal mediation effect (ACME), average direct effects (ADE), and total effects, are significant (*p* < 0.05) with the ACME estimate being 0.23 and the ADE estimate being −0.43.

### 3.4. Association of Calcium and BMD with Fracture

We took daily calcium intake as the independent variable, total BMD as the mediator, and hip fracture as the dependent variable. When including other covariates, we found that calcium intake is not significantly associated with hip fracture, so we excluded the covariates from the model and directly used daily calcium intake and total BMD in the model. The odds ratio indicates that the odds of having a hip fracture are 0.04 times less for participants having a unit of increase in total BMD. The results can be seen in Table 5; almost no mediation and direct effect could be found from the model.

## 4. Discussion

The current study mainly focuses on confirming and exploring a clear relationship and association between varied factors related to hip fracture status.

From the analysis between calcium intake and total BMD and femur BMD, we can see that the association between calcium intake and BMD is significant, which is in accordance with the common-sense notion that the more calcium we consume, the more our BMD level should increase [28]. However, unsurprisingly, from the analysis between BMD and hip fracture, there is also a strong connection between BMD level and hip fracture [29]. From the above, based on the large scale of the NHANES data, we can be confident that the intake of calcium was related to the increase in BMD level. Both the total BMD and femur BMD will increase by 0.04 units if we take an additional 1 g of calcium daily, which is consistent with the minimum 1000 mg/day recommendation of supplementary calcium intake for postmenopausal women [30,31]. Moreover, a 0.04 unit increase in BMD could result in around a 10% decrease in hip fractures based on our results. All of the above findings provide significant yet indirect proof that if we take more calcium daily, then eventually, with increasing BMD, the occurrence of hip fracture will go down.

Based on the above information, we then tried to establish a direct model with all these variables by using a mediation analysis model. Interestingly, mediation analysis using two different models provides some unexpected information. First, although a significant result was obtained when we treated femur BMD as the mediator and total BMD as the independent variable, the inconsistent mediation effect of femur BMD was significant [32], which means the direct and indirect effects are opposite in nature. This is basically because the direct effect is larger than the total effect, meaning that the total BMD level solely impacts the probability of hip fracture more than the total and femur BMD levels combined. A possible explanation is that the total BMD level is more important than the femur BMD level when considering the possibility of hip fracture. Additionally, while from various reports, we can see that the recommendation of supplementary calcium intake was included in the treatment and presentation of different conditions that lead to hip fracture [33,34,35], our mediation model that takes calcium intake as the independent variable and total BMD as the mediator variable does not produce a significant result for all effects. This might indicate that the calcium intake itself cannot strongly influence the probability of hip fracture directly, but taking more calcium will help increase the BMD level. With the increase in BMD level, the probability of hip fracture could be reduced. This may be because there are multiple causes that might lead to hip fracture, and BMD level is only one of them, while calcium intake might be associated with not only BMD level but also other factors, which means that calcium intake can have both positive and negative impacts on the occurrence of hip fracture.

As mentioned in other study, vitamin D could also be an important factor for preventing fractures [17,18]. Although vitamin D has not been included in the study due to the data source limitation, we know for long that vitamin D plays a critical role in various medical conditions not only for fracture we focused in this study but also for cancer, graft-versus-host disease, multiple sclerosis and epilepsy [36,37,38,39]. The pathway of how vitamin D functions have also been discussed a lot and recent studies have demonstrated that the vitamin D level influences on the level of pro-inflammatory cytokines (IL-6 and TNF-α) as well as the activity of T and B cells, which explains why vitamin D could contribute to hip fracture not only from calcium absorption but also from modulating inflammation and immune function [37,39]. The synergy effect of vitamin D and calcium could be further studied later with more data acquired [38].

There are several aspects to the limitations of this study. From 18,003 participants’ NHANES data, only 259 participants reported hip fractures in the survey, making the analysis less simple [40,41]. Moreover, the reported outcomes of hip fractures were collected retrospectively and reported by each participant, which means the time sequence of the outcome and the influencing factors are not necessarily in a reasonable order, and the quality of the data source is uncertain. Additionally, the cross-sectional nature of the NHANES study restricts the ability to establish causality, as the associations observed in the study cannot be used to confirm a temporal relationship between variables. Although multiple covariates were adjusted for the analysis, the possibility of unaccounted and unmeasured factors, such as vitamin D, physical activity, hormonal status, and genetic predisposition, impacting the results cannot be ruled out. Lastly, missing data bias may have affected the findings. All of these issues undermine the strength of the evidence level of this study [42,43]. Still, obtaining large amounts of data is hard for this kind of study; thus, we attempted to include all useful NHANES data and related references to support the analysis and try to identify the association in this study.

## 5. Conclusions

In conclusion, mediation analyses were used here to test whether there is a causal relationship between calcium intake, BMD level, and hip fracture. The NHANES data did not provide a clear causal relationship between all of these variables, but respectively, calcium intake and BMD level, as well as BMD level and hip fracture, have shown strong associations, which means that calcium intake influenced BMD level and BMD level had an impact on the occurrence of hip fracture, while the direct causal effect model could not yet be established among all of these three factors. In light of these observations, the supplementation of calcium could be recommended for maintaining high BMD levels. Future studies on more covariates and randomized studies could also be conducted to identify the clear relationship if possible.

## Figures and Tables

**Table 1:** Characteristics of study population.

	with Fracture (*n* = 259)	Without Fracture (*n* = 17,744)	Overall (*n* = 18,003)	*p*-Value ^c^
Sex				
Male	124 (47.9%)	8980 (50.6%)	9104 (50.6%)	0.418
Female	135 (52.1%)	8764 (49.4%)	8899 (49.4%)	
Age ^a^ (years)				
Mean (SD)	63.5 (14.6)	54.7 (16.3)	54.8 (16.3)	<0.001
Median [Min, Max]	64.0 [21.0, 85.0]	56.0 [20.0, 85.0]	56.0 [20.0, 85.0]	
Race				
Mexican American	25 (9.7%)	2724 (15.4%)	2749 (15.3%)	<0.001
Non-Hispanic Black	43 (16.6%)	3535 (19.9%)	3578 (19.9%)	
Non-Hispanic White	157 (60.6%)	8496 (47.9%)	8653 (48.1%)	
Other Race	34 (13.1%)	2989 (16.8%)	3023 (16.8%)	
Marital Status ^b^				
Married	94 (36.3%)	8539 (48.1%)	8633 (48.0%)	<0.001
Other	165 (63.7%)	9205 (51.9%)	9370 (52.0%)	
Family PIR				
Mean (SD)	2.33 (1.64)	2.70 (1.62)	2.69 (1.62)	<0.001
Median [Min, Max]	1.77 [0, 5.00]	2.38 [0, 5.00]	2.36 [0, 5.00]	
Missing	15 (5.8%)	1466 (8.3%)	1481 (8.2%)	
BMI (kg/m^2^)				
Mean (SD)	27.9 (6.67)	28.6 (5.76)	28.6 (5.78)	0.0892
Median [Min, Max]	26.7 [15.4, 51.8]	27.9 [13.2, 65.5]	27.9 [13.2, 65.5]	
Missing	1 (0.4%)	57 (0.3%)	58 (0.3%)	
Waist Circumference (cm)				
Mean (SD)	98.8 (16.4)	99.0 (14.4)	99.0 (14.4)	0.885
Median [Min, Max]	97.5 [62.0, 154]	98.4 [57.9, 164]	98.3 [57.9, 164]	
Missing	3 (1.2%)	132 (0.7%)	135 (0.7%)	
Smoking ᵇ				
Ever	157 (60.6%)	8336 (47.0%)	8493 (47.2%)	<0.001
Other	102 (39.4%)	9408 (53.0%)	9510 (52.8%)	
Drinking ᵇ				
Ever	30 (11.6%)	1833 (10.3%)	1863 (10.3%)	0.579
Other	229 (88.4%)	15,911 (89.7%)	16,140 (89.7%)	
Diabetes Status ᵇ				
Yes	51 (19.7%)	2481 (14.0%)	2532 (14.1%)	0.0113
Other	208 (80.3%)	15,263 (86.0%)	15,471 (85.9%)	

ᵃ Age at screening; ᵇ other includes all other statuses; ^c^ for numeric variables, we performed *t*-tests; for categorical variables, we performed chi-squared tests; BMI, body mass index; Family PIR, ratio of family income to poverty.

**Table 2:** The associations between calcium (g) and BMD.

Dependent Variable	Characteristic	Estimate	95% CI ^1^	*p*-Value
Total BMD	Daily Calcium Intake (g)	0.04	0.04, 0.05	<0.001
Femur BMD	Daily Calcium Intake (g)	0.04	0.03, 0.04	<0.001

^1^ CI = confidence interval.

**Table 3:** The associations between BMD and fracture.

Characteristic	log(OR) ^1^	95% CI ^1^	*p*-Value
Total BMD	−5.4	−7.2, −3.6	<0.001
AGE	0.02	0.01, 0.03	<0.001
DRINK			
Ever	—	—	
Other	−0.38	−0.64, −0.11	0.005
PIR	−0.10	−0.19, −0.02	0.016
MARRIAGE			
Married	—	—	
Other	0.32	0.06, 0.59	0.018
SEX			
Male	—	—	
Female	−0.35	−0.64, −0.07	0.016
Femur Head BMD	2.6	0.55, 4.5	0.011
DIABETES			
Yes	—	—	
Other	−0.34	−0.64, −0.01	0.037
RACE			
Mexican American	—	—	
Non-Hispanic Black	0.13	−0.37, 0.65	0.6
Non-Hispanic White	0.43	0.00, 0.89	0.058
Other Race	0.10	−0.42, 0.64	0.7

^1^ OR = odds ratio, CI = confidence interval.

**Table 4:** Femur BMD as a mediator in the associations between hip fracture and total BMD.

Characteristic	Estimate	95% CI ^1^	*p*-Value
Total Effect	−0.20	−0.38, −0.09	<0.001
ACME (average)	0.23	0.04, 0.34	0.010
ADE (average)	−0.43	−0.62, −0.21	<0.001
Prop. Mediated (average)	−1.1	−2.8, −0.17	0.010

^1^ CI = confidence interval.

**Table 5:** Total BMD as a mediator in the associations between hip fracture and calcium intake.

Characteristic	Estimate	95% CI ^1^	*p*-Value
Total Effect	0.00	−0.01, 0.01	0.8
ACME (average)	0.00	0.00, 0.00	>0.9
ADE (average)	0.00	−0.01, 0.01	0.8
Prop. Mediated (average)	0.00	0.00, 0.00	>0.9

^1^ CI = confidence interval.

## Data Availability

The datasets presented in this study can be found in online repositories. The names of the repository/repositories and accession number(s) can be found below: https://www.cdc.gov/nchs/nhanes/ (accessed on 10 December 2024). The generated data using for the study can be found from the Supplementary Materials.

## Supplementary Materials

The following supporting information can be downloaded at https://www.mdpi.com/article/10.3390/healthcare13040356/s1, Data: generated data for the analysis of the study.
